# Chemical-Vapor-Deposition-Synthesized Two-Dimensional Non-Stoichiometric Copper Selenide (β-Cu_2−x_Se) for Ultra-Fast Tetracycline Hydrochloride Degradation under Solar Light

**DOI:** 10.3390/molecules29040887

**Published:** 2024-02-17

**Authors:** Srijith, Rajashree Konar, Eti Teblum, Vivek Kumar Singh, Madina Telkhozhayeva, Michelangelo Paiardi, Gilbert Daniel Nessim

**Affiliations:** 1 Department of Chemistry, Bar-Ilan Institute of Nanotechnology and Advanced Materials, Bar-Ilan University, Ramat Gan 5290002, Israel; srijith.nitk@gmail.com (S.); rajashree.konar@biu.ac.il (R.K.); eti.teblum@gmail.com (E.T.); vivekkumarsingh.rs.cer18@itbhu.ac.in (V.K.S.); telkhozhayeva@gmail.com (M.T.); 2 Department of Chemistry and Materials Engineering “Giulio Natta”, Politecnico Di Milano, Piazza Leonardo da Vinci, 32, 20133 Milano, Italy; michelangelo.paiardi@mail.polimi.it

**Keywords:** 2D materials, photocatalysis, chemical vapor deposition, copper selenide, antibiotic, degradation

## Abstract

The high concentration of antibiotics in aquatic environments is a serious environmental issue. In response, researchers have explored photocatalytic degradation as a potential solution. Through chemical vapor deposition (CVD), we synthesized copper selenide (β-Cu_2−x_Se) and found it an effective catalyst for degrading tetracycline hydrochloride (TC-HCl). The catalyst demonstrated an impressive degradation efficiency of approximately 98% and a reaction rate constant of 3.14 × 10^−2^ min^−1^. Its layered structure, which exposes reactive sites, contributes to excellent stability, interfacial charge transfer efficiency, and visible light absorption capacity. Our investigations confirmed that the principal active species produced by the catalyst comprises O^2−^ radicals, which we verified through trapping experiments and electron paramagnetic resonance (EPR). We also verified the TC-HCl degradation mechanism using high-performance liquid chromatography–mass spectrometry (LC-MS). Our results provide valuable insights into developing the β-Cu_2−x_Se catalyst using CVD and its potential applications in environmental remediation.

## 1. Introduction

Antibiotics have been widely used in recent years for medicinal and non-medicinal purposes in hydroponics, animal husbandry, agriculture, and human therapies, such as treating infectious diseases like COVID-19, transplants, chemotherapy, and surgical interventions [1,2,3]. However, due to improper use in the pharmaceutical industry, homes, farms, and fisheries continuously release antibiotics into water bodies. Due to these issues, antibiotics have become significant emerging contaminants (ECs) in water, posing a substantial threat to human health. Antibiotic molecules have a complex structure, such as the linear fused tetracyclic nucleus comprised of four rings in tetracyclines. These molecules have antibacterial properties that make it difficult for microorganisms to break them down in the environment. Tetracyclines (TCs) exhibit long half-lives, with a half-life of 4.15 days [4]. As a result, these compounds can travel several kilometers downstream of an average river before reaching a concentration equal to 50% of their original levels [5]. Antibiotics must, therefore, be eliminated from the water environment as soon as possible. Removing TCs from water using photocatalytic oxidation is promising, cost-efficient, and sustainable [6]. Mainly, photocatalytic degradation is a powerful reactive-species-based technique. Various antibiotics may be broken down by hydroxyl radicals (OH•) and superoxide anions (O^2−^•), converting them into less harmful organics while generating a low-molecular intermediate product: CO_2_ and H_2_O [7,8].

Despite extensive research in this field in recent years, the performance of photocatalysts is limited by certain limitations [9]. For example, the recombination of electro–hole pairs occurs during the charge transfer process because of the low mobility of photo-induced charge carriers in semiconductors, and it becomes worse when surface reactions cannot promptly consume the charge carriers because of the low activity and small number of catalytic sites on semiconductor surfaces [10]. The extreme charge recombination significantly reduces the quantum efficiency of photocatalysis. Due to exceptional chemical and physical properties, photocatalysts made from two-dimensional (2D) materials have drawn increased interest from researchers, especially those with various morphologies [11]. Numerous 2D materials, including transition metal oxides (TMOs) [10,12], transition metal dichalcogenides (TMDCs) [13,14,15], metal–organic frameworks (MOFs) [16,17,18], black phosphorous [19], and various metal nitrides (MNs) [20,21], are widely studied for their photocatalytic applications. Among these photocatalytic materials, 2D transition metal dichalcogenides (TMDCs) offer a fascinating material library [22]. Their atomic nature, spin orbit, tunable band gap, direct energy gaps in monolayer counterparts in the near-infrared to a visible region, strong excitonic effects, and naturally abundant nature have made these materials promising in photocatalysis applications [23,24,25,26]. In this regard, copper chalcogenides and copper oxide nanostructures have become interesting materials for photodegradation because of the optical characteristics derived from their crystalline phases. Their exceptional characteristics have made them highly desirable as photocatalysts in recent years [27,28,29]. The molar ratio of chalcogens/Cu affects the properties and phase of the material. For example, copper chalcogenide nanostructures with various phases have different properties and applications [30,31]. Other studies also indicate that the Cu_2_Se and Cu_3_Se_2_ phases of copper selenide can be used in solar cells as counter electrodes to increase the efficiency of cells [32,33,34]. In addition, Cu_2_S and Cu_3_Se_2_ are also reported as good photocatalysts materials for degrading pollutants from wastewater [35,36,37]. Similarly, diverse copper chalcogenide defects and phases result in the development of various optical and electrical characteristics. By appropriately modifying the phases and composites of copper selenides, we can tune the band gap (1.5–2.5 eV) [38,39,40], which is important for photocatalytic material to remove pollutants under visible-light-source irradiation.

We report the synthesis, characterization, and, to the best of our knowledge, for the first time, photocatalytic activity of chemical-vapor-deposition (CVD)-grown, non-stoichiometric copper selenide (β-Cu_2−x_Se) towards the removal of the antibiotic tetracycline hydrochloride (TC-HCl). As mentioned earlier, several reports of copper selenide phases acting as successful photocatalysts towards different organic dyes and pollutants. However, we did not find reports for TC-HCl degradation using β-Cu_2−x_Se. We detailed an optimized process to synthesize phase-pure monoclinic β-Cu_2−x_Se on Cu foil using an ambient pressure chemical vapor deposition (AP-CVD) system. β-Cu_2−x_Se is an effective catalyst for the ultra-fast degradation of the antibiotic TC-HCl without the need for any additional co-catalyst, dopant, or noble metal for functionalization. Our photocatalysis experiments reveal that powdered β-Cu_2−x_Se shows excellent ultra-fast degradation of TCH-HCl (~98.3%) following pseudo-first-order kinetics (where k_Cu2−xSe_ = 3.14 × 10^−2^ min^−1^). We will provide insights into the underlying mechanisms of this process and highlight the potential of β-Cu_2−x_Se as a promising catalyst in sustainable and eco-friendly wastewater treatment. The superior photocatalytic performance can be attributed to the catalyst’s layered structure and increased availability of reactive sites. EPR (electron paramagnetic resonance) confirmed that the high performance of the catalyst is due to the generation of the •O^2−^ radical.

## 2. Results and Discussion

### 2.1. Understanding the Microstructural Growth, Phase Purity, Thickness, and Related Characterizations of β-Cu_2−x_Se

Copper and selenium can combine in different ways to form stoichiometric and non-stoichiometric phases, such as cubic berzelianite (Cu_2_Se, Cu_1.8_Se, and Cu_2−x_Se), hexagonal klockmannite (CuSe and Cu_0.87_Se), tetragonal umangite (Cu_3_Se_2_), and orthorhombic athabascaite (Cu_5_Se_4_ and CuSe) [41]. Cu_2_Se possesses a β-phase (monoclinic structure) with the space group of C2/c at low-temperature and a high-temperature α-phase (cubic structure) with the space group of Fm-3m, and the exact transition temperature depending on the degree of copper deficiency. Prior studies have demonstrated the growth of copper selenide in bulk and its microstructural changes on Cu foils [42,43]. Most reports in this field emphasize the solvothermal-based routes for copper selenide growth. However, limited research is devoted to the CVD-based synthesis of copper selenide on Cu foil. Furthermore, the reaction mechanism for this process has not been thoroughly explored. To analyze the growth process, we started with the copper selenium phase diagram to understand the different phases attributed to changes in temperature and precursor quantities [44]. Our current work focused on the synthesis and microstructural control of Cu_2−x_Se using chemical vapor deposition, despite the significant research on copper selenide synthesis.

**Figure 1:**
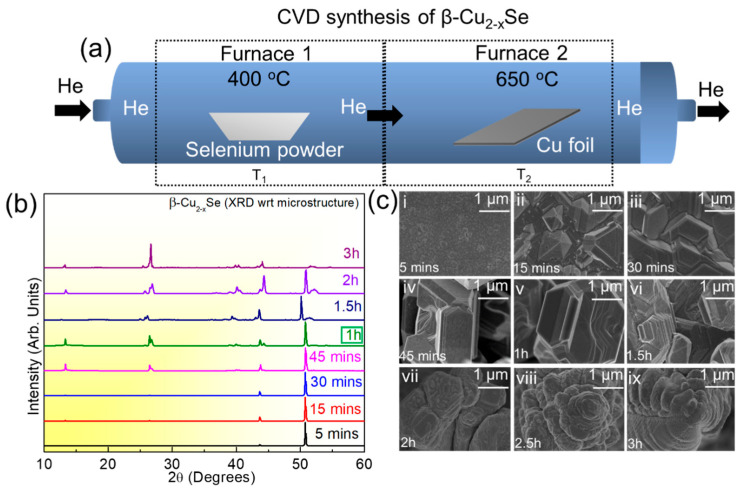
(**a**) Schematic for the chemical vapor deposition (CVD) two-furnace setup to synthesize and optimize copper selenide (Cu_2-x_Se) on Cu foil. The elemental precursors were kept in two different furnaces. Furnace 1 or F1 contained the Se powder at 400 °C, and Furnace 2 or F2 had the acid-etched Cu foil at 650 °C; (**b**) X-ray diffractogram (XRD) variation in copper selenide growth on Cu foil; (**c**) high-resolution scanning electron microscopy (HRSEM) showing microstructural evolution and formation of copper selenide at different reaction times (5 min to 3 h) between etched Cu foil and elemental Se.

The fine-tuning of the reaction parameters to synthesize copper selenide on Cu foil is akin to the protocols we have reported previously [45,46,47]. We used a two-furnace setup in series, as shown in the schematic in Figure 1a. We tuned the reaction time of thermal annealing to obtain uniform coverage or pure-phase material on the exposed surface of the Cu foil and to check its morphology, structure, and purity. After the reaction between Cu foil and elemental Se was completed, the cooling rates were varied to form the β-Cu_2−x_Se phase. Variations in the cooling rates were performed at (i) 50 °C per minute, (ii) 10 °C per minute, and (iii) 5 °C per minute. It is observed that the slower cooling rate of 5 °C per minute led to the formation of a phase-pure copper selenide compound in comparison to the other two cooling rates, which led to the formation of a bi-phasic copper selenide. We also analyzed the role of the surface etching of the Cu foils using acid-etched and unetched Cu foils in the same quartz boat as the control. The effect of acid etching on the Cu foil’s surface proved beneficial to its growth, as the unetched Cu foils did not show any observable growth for the same reaction conditions.

We optimized the CVD synthesis of copper selenide based on reaction temperatures, reaction times, amount of elemental precursors, and carrier gases. The sublimation of elemental selenium (0.5 g) was fixed at 400 °C in F1, as this temperature was most suitable to obtain the desired Se vapor pressure for the reaction [48,49]. The Cu foil temperature was varied between 500 and 700 °C. Of all the Cu foil temperature variations, 650 °C was most helpful in synthesizing β-Cu_2−x_Se with good crystallinity, large flake sizes after liquid-phase exfoliation, and phase purity. The X-ray diffractogram (XRD) variation in copper selenide growth on Cu foil at different reaction times from 5 min to 3 h is shown in Figure 1b. High-resolution scanning electron microscopy (HRSEM) measurements show the microstructural growth for the different reaction times (Figure 1c). Our studies show that the layer-by-layer bulk growth of the copper selenide starts from the random nucleation sites formed on the etched Cu foil after solid–vapor interaction with the sublimated Se (ex situ HRSEMs are shown in Supplementary Figure S1a–i). The microstructural evolution of the bulk β-Cu_2−x_Se as a function of reaction time (keeping all other parameters constant) shows the gradual formation of β-Cu_2−x_Se stacks on top of each other. As a result, the microstructure with a precise number of layers on top of each other was only observed for the 1 h reaction (which was also the same optimization as reported in our previous works) [45,46,47,50,51]. For reaction times lower than 1 h (5 min to 45 min), we obtained flakes that were hard to exfoliate or scratch from the Cu foil surface. Reaction times greater than 1 h led to a dendritic β-Cu_2−x_Se formation, which was unsuitable for our photocatalysis measurements. The growth details as a function of large-area HRSEM analysis are shown in Supplementary Figure S1a–i. Electron dispersive spectroscopy (EDS) and area mapping analysis were used to confirm the Cu and Se content at each reaction time, as shown in Supplementary Figure S2 (1–31). We also determined a probable reaction mechanism for the growth and evolution of β-Cu_2−x_Se on Cu foil and show it in Supplementary Figure S3.

We also varied the carrier gases and observed that helium produced highly crystalline β-Cu_2−x_Se. The helium flows were optimized from 100 to 150 sccm. The long purging time (30 min with 500 sccm of helium) ensured that the quartz tube was completely oxygen-free. No reducing atmosphere was used to ensure that toxic byproducts of H_2_Se formation could be eliminated during the synthesis protocol. The CVD route we reported in this paper has a significant advantage compared to other processes. We have previously reported on the CVD route’s reproducibility and scalability, especially when using transition metal foils as the growth substrate [45,46,47,51,52,53]. We have started our synthesis from elemental precursors, i.e., Cu foil and Se powder. We selected 650 °C and 400 °C for the Cu foil zone and Se powder zone by extracting information related to the different phases reported in the Cu-Se phase diagram. The entire process is repeatable, simple, and scalable. The crystallinity and phase purity of β-Cu_2−x_Se are preserved and maintained even when scaling up the process (it can be upscaled up to 2 g of β-Cu_2−x_Se per CVD synthesis).

**Figure 2:**
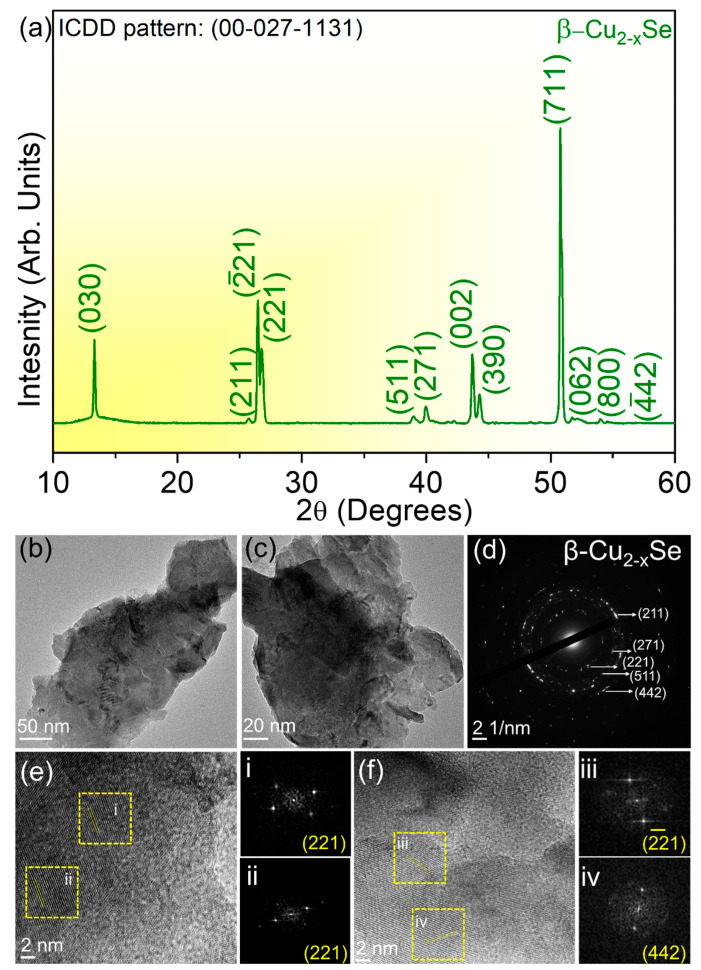
(**a**) X-ray diffractogram (XRD) confirming the β-Cu_2-x_Se monoclinic phase-pure structure. All planes are indexed to ICDD #00-027-1131. (**b**,**c**) Bright field low magnification transmission electron microscopy (TEM) images showing the layers in monoclinic β-Cu_2-x_Se. (**d**) Selected area diffraction (SAED) pattern of monoclinic β-Cu_2-x_Se; (**e**,**f**) high-resolution transmission electron microscopy (HRTEM) images at 2 nm, indicating that stacking faults are also present in the material with the corresponding Fourier transforms (FFTs) in (i–iv).

Apart from the microstructural analysis, we determined that our growth protocol could form phase-pure monoclinic β-Cu_2−x_Se on Cu foil, as shown in Figure 2a. The as-synthesized β-Cu_2−x_Se corresponds to the ICDD pattern number 00-027-1131. The peaks in the bulk material can be assigned as follows.

(030) at 2θ~13°, (211) at 2θ~25.3°, (−221) at 2θ~26.3°, (221) at 2θ~26.4°, (511) at 2θ~38.8°, (271) at 2θ~39.8°, (002) at 2θ~43.6°, (390) at 2θ~44.2°, (711) at 2θ~50.8°, (002) at 2θ~43.6°, (062) at 2θ~51.5°, (800) at 2θ~51.8°, (−442) at 2θ~54.2°

**Figure 3:**
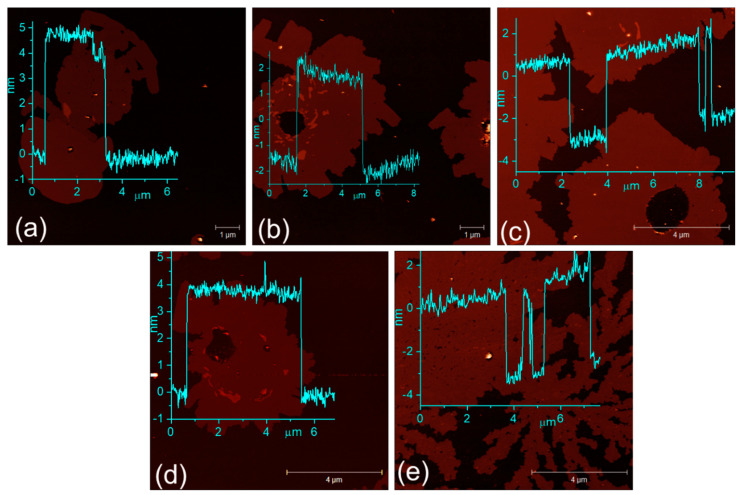
Atomic force microscopy (AFM) images of β-Cu_2-x_Se dispersed in absolute ethanol at 80 kHz 100% power. After dropcasting on Si/SiO_2_ wafers, the observed heights of the flakes are (**a**) 4.9 ± 0.3 nm, (**b**) 2.5 ± 0.3 nm, (**c**) 4.2 ± 0.3 nm, (**d**) 4.1 ± 0.3 nm, and (**e**) 3.9 ± 0.3 nm. All β-Cu_2-x_Se flakes are measured from random Si/SiO_2_ substrate areas.

Figure 2b–f show the transmission electron microscopy (TEM) images of the copper selenide sample. Since copper selenide was grown on a copper foil, although we would expect that XRD or TEM measurements might show some minute traces of pure copper during its assessment, we did not observe any pure copper. The bright-field low-magnification TEM images in Figure 2b,c show the clear presence of layers after exfoliating the material from two different areas on the Au TEM grid. The layered nature of the exfoliated β-Cu_2−x_Se is further confirmed via the atomic force microscopy (AFM) images, as shown in Figure 3a–e. The selected area diffraction (SAED) pattern is taken from the Cu_2−x_Se particle. The presence of (211) at d = 0.3507 nm, (271) at d = 0.226 nm, (221) at d = 0.2646 nm, (511) at 0.231 nm, and (442) at 0.168 nm is a clear indication of the material belonging to the monoclinic phase of β-Cu_2−x_Se. Also present in the sample are 2D stacking faults, as evidenced by the high-resolution transmission electron microscopy (HRTEM) images in Figure 2e,f. The corresponding fast Fourier transforms (FFTs) from Areas (i)–(iv) are also shown in Figure 2. Several structures for β-Cu_2−x_Se have been suggested in the literature, but they disagree with the measured scattering data. One such study used the 3D-PDF analysis method for diffuse single-crystal X-ray scattering and revealed that β-Cu_2−x_Se’s structure comprises two-dimensional ordered layers stacked in a highly disordered sequence [54]. Two modes of stacking disorder manifest in mirrored and non-mirrored layers and the sequence of three possible inter-layer vectors [54]. The resulting monoclinic structure has a higher average symmetry due to the disordered stacking [54]. Most proposed theoretical models and experimental findings for Cu-Se crystal systems assume a periodic structure in three dimensions. Still, the monoclinic structure reported here is ordered in only two dimensions, with stacking disorder in the third dimension (The detailed structural analysis is also reported in Ref. [54]). Many suggested structures are also periodic repetitions of structural units that are found in the real disordered structure [55,56], where the authors have suggested monoclinic and triclinic structures, while others have proposed structures based on DFT calculations. In particular, the triclinic structure has the stacking of identical layers, whereas the monoclinic structure has the stacking of alternating mirrored and non-mirrored layers [57]. These structures have relatively small energy differences, and the stacking disorder found in the real structure is likely due to the almost identical enthalpy of the different stacking types and a higher entropy term from the disorder [55]. Several of the suggested structures found in the literature have the correct layer types, but periodic stacking sequences were noted to have very similar enthalpies of formation. In our work, we have seen the stacking faults arising from the copper selenide layers, and we hypothesize that it is responsible for the enhanced photocatalytic activity under simulated solar light. Since the photocatalytic performance of Cu_2−x_Se nanocrystals is limited by the high recombination rate of the photogenerated free carriers [56], the deficiency of copper ions generates hole carriers in the monoclinic β-Cu_2−x_Se system, decreasing the electrical resistivity. As indicated in a previous study, the carrier concentration and mobility at room temperature of β-Cu_2−x_Se are significantly larger than those of α-Cu_2−x_Se [55].

The Raman spectrum using a 532 nm laser for bulk β-Cu_2−x_Se is around 262 cm^−1^, and for exfoliated β-Cu_2−x_Se, it is around 259 cm^−1^, as shown in Supplementary Figure S4b. The semiconducting nature of monoclinic β-Cu_2−x_Se was estimated via experimentally obtained bandgap values. The bandgap value, calculated from the Tauc plot, also agrees with the reported data in previous works [58,59]. 

To further investigate the energy band structure of β-Cu_2−x_Se, we measured the UV-visible diffuse reflectance, and the data were analyzed using the Kubelka–Munk function to obtain the band gap (*E_g_*), as shown in Equation (1), below:

(1) $${\left(\alpha h\nu \right)}^{1/n}=A(h\nu -E_{g})$$

**Figure 4:**
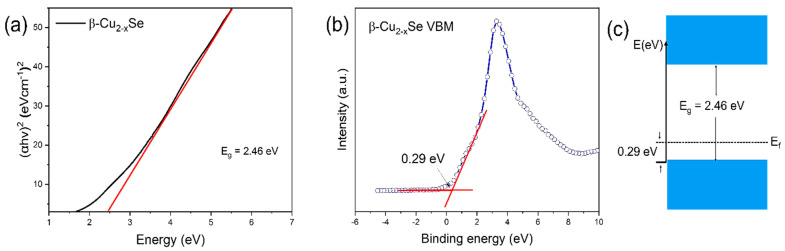
(**a**) Bandgap energy calculation for β-Cu_2−x_Se. (**b**) Valence-band XPS of β-Cu_2−x_Se. (**c**) Schematic sketch of the band positions in β-Cu_2−x_Se.

*α*, *h*, *ν*, and *A* denote the absorption coefficient, Planck constant, light frequency, and constant, respectively. The value of *n* depends on the semiconductor’s band gap. (*n* is 0.5 for a direct band gap, *n* is 2 for an indirect band gap). We calculated the bandgap, as shown in Figure 4a, by plotting (*αhν*)^2^ vs. *hν*. *E_g_*, is the value tangent drawn to the *x*-axis. Gáborová et al. discovered an emission peak at 2.56 eV and another at 2.36 eV in the photoluminescence spectra of Cu_2_Se due to a defect-related emission induced by copper vacancies and interstitial defects in the chemically prepared sample. Here, we obtained the bandgap energy of 2.46 eV from the above equation, and the value is almost the same as per previously reported findings [58,59]. We calculated the valence band maximum (VBM) values by intercepting two lines, as shown in Figure 4b. The VBM of the β-Cu_2−x_Se was calculated to be 0.29 eV. Figure 4c summarizes the energy level band structure of β-Cu_2−x_Se using computed *E_g_* and VBM values.

### 2.2. Photodegradation of Tetracycline Hydrochloride (TC-HCl)

**Figure 5:**
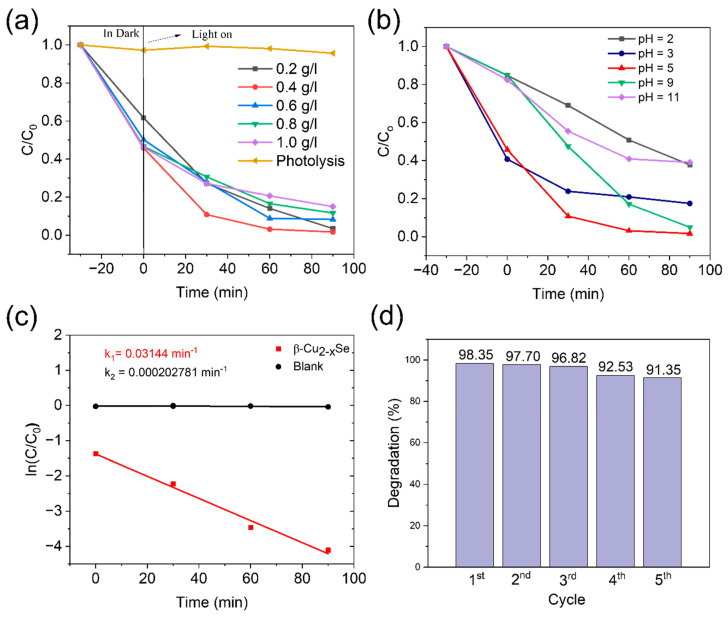
(**a**) Photocatalytic degradation of TC-HCl to time in the presence of different loading of β-Cu_2-x_Se under simulated solar light irradiation. (**b**) Effect of solution pH. (**c**) TC-HCl degradation kinetics. (**d**) Cycling test of β-Cu_2−x_Se.

We evaluated the photodegradation of TC-HCl by β-Cu_2−x_Se in the presence of simulated solar light irradiation. As shown in Figure 5a, with increasing irradiation time, TC-HCl concentration remained practically unchanged in the absence of β-Cu_2−x_Se. We performed the photodegradation of TC-HCl with different loading of the catalyst. The oxidation capacity linearly increased as the amount increased from 0.2 g L^−1^ to 0.4 g L^−1^ (Figure 5a) because of the increased reactive activation sites caused by the photocatalyst [60]. The further increase in loading (0.6 g L^−1^ to 1.0 g L^−1^) showed a reduction in photocatalytic degradation (98.3 to 85%). The turbidity of suspensions, light scattering, and the accumulation of solid particles increase with an increase in catalyst concentration. This excess particle screening action covers some of the photosensitive surfaces. Consequently, fewer catalyst particles may be activated and the photon penetration depth falls. As a result, the rate of degradation drops [61]. The pH of the reaction affects the generation of reactive oxygen species on the surface of the photocatalyst material, which affects the degradation activity of pollutants. Usually, TC-HCl exists in three forms (TCH^+^, TCH^0^, and TCH^−^) at distinct pHs (pH ˂ 4.4 ˂ pH ˂ 7.5, and pH > 7.5) [62,63].

Here, the pH value of the original TC-HCl solution was 5, and we altered the pH using 0.1 M HCl and NaOH to check the effect at different PHs. Figure 5b shows that the degradation efficiency considerably decreased in highly acidic conditions (pH = 2.3). The decrease in pH may be attributed to the enhanced repulsive force between the TCH^+^ and the positively charged catalyst. The catalyst displayed better adsorption capacity in alkaline conditions, implying that the surface complexation was more intense than the repulsive force. This force played a significant role in absorbing TC-HCl [64]. However, when the pH was adjusted from 9 to 11, the degradation efficiency dropped from 95% to 60.9%. This is due to increased antibiotic adsorption suppressing photon utilization. Furthermore, redundant OH could deplete partial •OH and h^+^ [65]. The results showed that weak acid or weak alkali conditions were more favorable for TC-HCl photodegradation. Therefore, pH 5 was chosen as an optimal condition in all experiments.

Figure 5c depicts the rate constant of the β-Cu_2−x_Se performance versus blank. To quantify our catalyst’s photoactivity, we used the pseudo-first-order kinetic model to estimate the reaction rate constants of TC-HCl degradation, as shown in Equation (2), below:

(2) $$\ln\left(\frac{C}{C_{0}}\right)=kt$$

where *k* is the apparent reaction rate, and *t* is the irradiation time. By fitting the experimental data to the pseudo-first-order, the kinetics of TC-HCl degradation were assessed, and we obtained k_1_ = 3.14 × 10^−2^ min^−1^ and k_2_ = 2.02 × 10^−4^ min^−1^.

The reusability of the photocatalyst is also a significant factor in real-world photocatalytic applications. We tested the stability of β-Cu_2−x_Se using four cycles of TC-HCl photodegradation, as shown in Figure 5d. After each cycle, the sample was filtered, washed, and dried. Following the first, second, third, fourth, and fifth cycling runs, the percentage of TC-HCl degradation remained at 98.35, 97.70, 96.82, 92.53, and 91.35%. This indicates that the β-Cu_2−x_Se efficiently prevented photo corrosion during the reaction and possessed long-term stability. The slight reduction in photocatalytic activity could be attributed to the inevitable bulk loss of the catalyst throughout each cycle test. We analyzed the shape and crystalline structure of β-Cu_2−x_Se following the photocatalytic reaction. HRSEM and TEM analysis demonstrated that the morphology of β-Cu_2−x_Se remained stable after four recycle operations (Supplementary Figure S5a,b). There was no observable phase change or degradation in the layered morphology of β-Cu_2−x_Se, as shown in Supplementary Figure S5c,d. The X-ray photoelectron spectroscopy (XPS) measurements of the pristine and cycled β-Cu_2−x_Se are shown in Supplementary Figure S6. The splitting of spin-orbit components in the Cu 2p and Se 3d peaks is evident from Figure S6a,b. The peaks are significantly split into Cu 2p_1/2_, Cu 2p_3/2_, Se 3d_3/2_, and Se 3d_5/2_ components. The binding energy of Cu 2p_3/2_ is at 933.2 eV, and for Cu 2p_1/2,_ it is seen at 952.4 eV. As reported in the literature, two satellite peaks of Cu are also observed, which is characteristic of the non-stoichiometric β-Cu_2−x_Se phase [66]: the Se 3d peaks, Se 3d_5/2_ at 53.7 eV, and Se 3d_3/2_ at 55.3 eV. Figure S6c,d show the XPS measurements of post-cycled β-Cu_2−x_Se that show similar binding values for Cu 2p and Se 3d components with a slight decrease in their intensities. These findings point to the stability of the CVD-synthesized β-Cu_2−x_Se after rigorous photocatalytic tests.

### 2.3. Photocatalytic Degradation Mechanism

**Figure 6:**
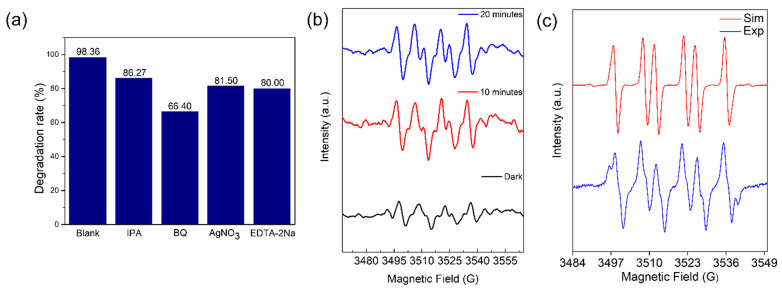
(**a**) Trapping experiments into the degradation of TC-HCl. (**b**) Electron paramagnetic resonance (EPR) of TC-HCl degradation by DMPO in a β-Cu_2−x_Se system. (**c**) Experimental and simulated EPR of the β-Cu_2−x_Se.

We conducted free radical trapping tests to determine the role played by each active species during the degradation of the TC-HCl. In this experiment, the scavengers of vacant electrons (ē), holes (h^+^), superoxide radicals (•O^2−^), and hydroxyl radicals (•OH) were silver nitrate (AgNO_3_,), ethylenediaminetetraacetic acid disodium salt (EDTA-2Na), 1,4-benzoquinone (BQ), and isopropyl alcohol (IPA). As shown in Figure 6a, the degradation rates in the presence of quenchers were 80% (EDTA-2Na), 81.50% (AgNO_3_), 86.27% (IPA), and 66.40% (BQ), respectively; these rates were significantly lower than the degradation rates without quenchers (98.36%). TC-HCl breakdown involves four active species: h^+^, ē, •O^2−^, and •OH. When •O^2−^ was collected by 1,4-benzoquinone (BQ), the degradation of TC-HCL decreased to 66.40%, showing that •O^2−^ predominated among them.

To further validate the existence of •O^2−^, electron paramagnetic resonance (EPR) was carried out utilizing 5,5-Dimethyl-1-pyrroline N-oxide (DMPO) as the spin-trapping agent. As shown in Figure 6b, the intense peaks were observed with light illumination, proving that a large number of reactive free •O^2−^ radicals can be formed during the photocatalytic reaction, which could aid in the TC-HCl degradation. In Figure 6c, we conducted experimental and simulated EPR spectra for the β-Cu_2−x_Se. The simulation parameters were established as g = 2.006, a_N_ = 13.9, and a_H_ = 10 for DMPO-OOH; g = 2.006, a_N_ = 14, and a_H_ = 12.7 for DMPO-OH; and g = 2.006, a_N_ = 16, and a_H_ = 22 for DMPO-CH2O. After 60 min of illumination, approximately 89% •O^2−^, 9% •OH, and 2% •CH_2_O were present.

**Figure 7:**
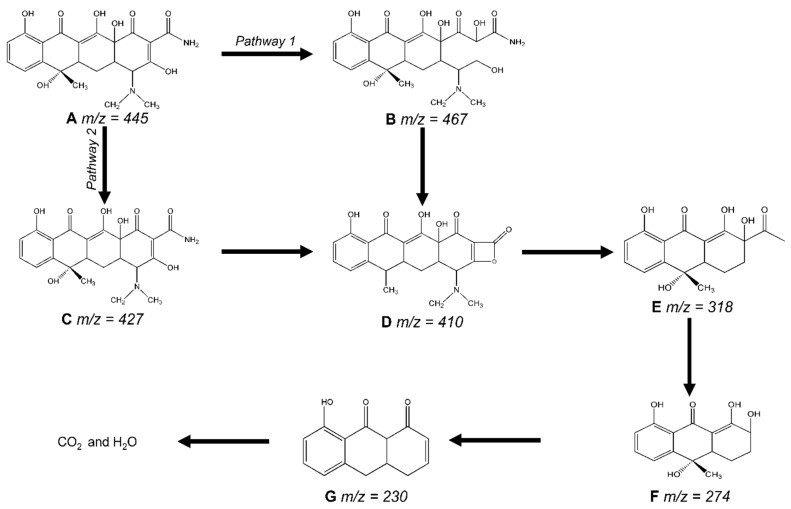
The proposed degradation pathway of TC-HCL by β-Cu_2−x_Se.

High-performance liquid chromatography (HPLC) assessed the β-Cu_2−x_Se photocatalytic degradation TC-HCl performance. Supplementary Figure S7 illustrates that the retention time for TC-HCl was 3.135 min. The process caused the TC-HCl characteristic peak’s intensity to drop gradually, showing that the substance had been mineralized and degraded. LC-MS techniques were employed for qualitative analysis based on the mass spectrum to determine the suggested TC-HCl degrading mechanism. In Supplementary Figure S7, the initial sample had a mass-to-charge ratio of 445 (*m*/*z*). The TC-HCl peaks were significantly reduced after exposure to visible light, and many new peaks with *m*/*z* values of 467, 427,410, 318, 274, and 230 were produced. We also display the mass spectra of TC-HCl transition products in Supplementary Figure S8. This suggests that TC-HCl was eventually transformed into lower-molecular-weight intermediates [67]. We predict that the breakdown mechanism of TC-HCl in the presence of β-Cu_2−x_Se and visible light is as shown in Figure 7, based on the findings, and when combined with earlier reports [67,68,69,70]. TC-HCl (*m*/*z* = 445) comprises double bonds, amines, and phenolic groups. For pathway 1, the ring-opening reaction caused the TC-HCl molecule to be oxidized by h^+^, •OH, and •O^2^ at the first stage of degradation, resulting in the intermediate product B (*m*/*z* = 467) [71]. For pathway 2, the loss of hydroxyl groups may have contributed to the intermediate product C (*m*/*z* = 427). The loss of C-NH_2_ was responsible for the product D (*m*/*z* = 410) [72,73]. With the following loss of carbon atom rings, the D was later broken down into E (*m*/*z* = 318). Product E was subsequently decarboxylated to produce products F (*m*/*z* = 276) and G (*m*/*z* = 230) [74]. These organic intermediates eventually underwent progressive mineralization to produce carbon dioxide, water, and other inorganic compounds. We also provide a comparison of our catalyst’s efficiency with that of other photocatalysts in the literature in Table S1.

## 3. Materials and Methods 

### 3.1. CVD Synthesis of β-Cu_2−x_Se 

All reagents were analytical grade and utilized in studies without further purification. We synthesized copper selenide (β-Cu_2−x_Se) using an atmospheric-pressure chemical vapor deposition (AP-CVD) system with two one-zone furnaces in series (Lindberg Blue), through which we heated a single fused silica (quartz) tube. The temperatures were measured using the thermocouples in a furnace (schematic is shown in Figure 1a). During the growth, the temperatures were set at 400 °C for the upstream furnace, which we will call F1, and at 650 °C for the downstream furnace, which we will call F2. The helium gas flow was controlled by a digital controller (MKS model P4B), and mass flow was measured by a mass flow control unit (MKS model 247D).

The synthetic procedure was as follows. Initially, we deposited 1 g of selenium powder (Alfa Aesar, 99.999%) in a boat tube outside the furnace, upstream of the gas flow. Then, we placed a second quartz boat with a 2 cm × 2 cm piece of acid-etched copper (Cu) foil (Alfa Aesar, 99.999%) downstream outside the heated zone of the furnace. The copper foil was etched before selenization with a 0.01 (M) HCL solution. Then, 2 cm × 2 cm Cu foil was purged with helium for 30 min to remove all air, while the two boats were kept at room temperature until the furnace reached equilibrium at the selected temperatures in the two zones. For the growth of the copper selenide, we pushed the quartz tube inside the furnace to position the copper (Cu) foil in the middle of F2 (at 650 °C). Using an external magnet, we introduced the selenium boat inside the furnace F1 (400 °C). At the end of the reaction, we first pulled out the selenium boat from F1. We allowed the sample at F2 to stay inside the furnace until it reached room temperature, under a constant flow of helium, before removing it from the furnace and exposing it to air. When we withdrew the finished product from the furnace, we discovered a thin black coating on the upper exposed surface of the copper foil. We varied the growth times of copper selenide (β-Cu_2−x_Se) among 1, 5, 15, 30, and 45 min. Extended growth times of 1, 2, and 3 h were also examined. Apart from preparing β-Cu_2−x_Se, we also compared its exfoliation compatibility in different solvents. For this purpose, we used absolute ethanol and isopropanol from Alfa Aesar. The dispersions were dropcasted on Si/SiO_2_ wafers to check their thicknesses via surface analyses in random areas on the wafer.

### 3.2. Characterizations of the Bulk and Exfoliated β-Cu_2−x_Se 

X-ray diffraction measurements were conducted on bulk copper selenide (Cu_2−x_Se) using a Rigaku Smartlab X-ray diffractometer. We analyzed a 1 cm × 1 cm square sample of bulk copper selenide. The data were collected in the 2θ range from 10° to 60°, with a step size and scanning rate of 0.005° and 0.5°/min. The X-ray generator was operated at 40 kV with Cu Kα radiation (λ = 1.542 Å). The crystallinity and purity of the as-synthesized copper selenide on copper foil were further analyzed using X’pert HighScore Plus software, and the corresponding peaks in the XRD were identified. The bulk copper selenide sample was subjected to high-resolution scanning electron microscopy (HRSEM) with the Magellan 400 FEI for additional evaluation. We conducted additional surface analysis studies using atomic force microscopy (AFM) measurements. This was accomplished using a Bio Fast Scan scanning probe microscope (Bruker AXS). All images were captured in the tapping position with a FastScan-B (Bruker) silicon probe (spring constant: 18 N/m). The cantilever’s resonance frequency was 1400 kHz (in air). The measurements were taken under natural conditions. The photographs were taken in the retrace direction, with a scan rate of 2.0 Hz. The photographs’ resolution was 512 samples per line. Gwyddion Analysis software was used to process AFM images. Before examining the photographs, the “flatting” and “planefit” tools were used on each. We performed a high-resolution transmission electron microscopy (HRTEM) investigation into the exfoliated Cu_2−x_Se in JEOL, JEM 2100F (working at 200 kV). We applied the dropcasting approach to a gold TEM grid after dispersing the copper selenide sample in ethanol at 80 kHz and depositing one drop on the grid. The UV-Vis-NIR absorption spectroscopy was performed using Shimadzu’s SolidSpec-3700. The extinction spectra were collected at 300–800 nm wavelengths with a 0.2 nm step using a 2 mL quartz vial with a 10 mm optical path length and an integral sphere in the measurement setup. The spectrum of the initial solvent served as a baseline for all Cu_2−x_Se solutions. The Raman spectra were obtained using a confocal Raman microscope (alpha300 R+, WiTec) with a 532 nm excitation laser and an incident power of 1 mW. The XPS measurements were performed with a Thermo Scientific Nexsa spectrometer. The materials were exposed to a soft X-ray source (about 1.5 KeV) under ultrahigh vacuum conditions (approximately 10^−10^ to 10^−9^ torr), and the emitted photoelectrons were measured. The electron paramagnetic resonance (EPR) measurements were performed using the Bruker EMX EPR 100d X-band Spectrometer. This spectrometer is a continuous wave (CW) device that operates at 9.5 GHz, 30 mm, with a central magnetic field of roughly 3300 G.

### 3.3. Photocatalytic Tests

The catalyst’s performance (β-Cu_2−x_Se ) was evaluated by degrading TC-HCl antibiotic using a free-standing solar simulator (model 10500). The solar simulator features a 150 W Xe arc lamp with a 1.5 G AM filter that covers UV, visible, and infrared wavelengths (400–1800 nm). The model collects energy from the lamp using a back reflector and a quick F/1 optical system, producing an output equivalent to more than three suns over smaller regions. One sun performance with uniformity of +/− 25% is possible for lighted fields up to 35 mm in diameter. In this instance, the optical system creates a 25 mm beam and an input of 90–250 V, 50–60 Hz. The photocatalytic experiments were carried out at room temperature. For conducting experiments, the TC-HCl solution (5 × 10^−5^ M) volume is 50 mL and the photocatalyst dosage is 20 mg (0.4 g L^−1^). After adding the catalyst, the suspension is agitated in the dark for 30 min to achieve TC-HCl adsorption–desorption equilibrium on the photocatalyst surface before turning on the light for 90 min. Every 30 min, 2–4 mL of solution was filtered through a 0.22 μm cellulose filter using a syringe. A UV-Vis spectrophotometer with a wavelength of 357 nm was used to measure the collected solution. Similarly, the control experiment was carried out with several catalyst loadings and without a catalyst to determine any potential direct photolysis of TC-HCl.

The pH of the TC-HCl solution was changed with NaOH and HCl (0.1 M) to examine the impact of the initial pH. The photodegradation procedure was repeated four times to explore the photocatalyst’s stability, reproducibility, and reusability. After each reaction, the photocatalyst was recovered for the next cycle, and the TC-HCl concentration was measured using a UV-Vis spectrophotometer.

### 3.4. Trapping Experiments

The following trapping reagents were employed to investigate the active species for TC-HCl photodegradation—silver nitrate (AgNO_3_), ethylenediaminetetraacetic acid disodium salt (EDTA-2Na), 1,4-benzoquinone (BQ), and isopropyl alcohol (IPA)—which act as scavengers for electrons (ē), holes (h^+^), superoxide radicals (•O^2−^) and hydroxyl radicals (•OH). The procedure is the same as the test for photocatalytic activity, except that scavengers are injected before light irradiation. Additionally, the presence of •O^2−^ and •OH radicals was determined via EPR employing the spin-trapping agent 5,5-dimethyl pyrroline-N-oxide (DMPO, Sigma-Aldrich). The DMPO was filtered after being soaked in activated charcoal for 30 min in the dark; its concentration was then measured spectrophotometrically, yielding ε_227nm_ = 8.0 mM^−1^ cm^−1^. The generated spectra were recorded at microwave frequencies of approximately 9.8 GHz, microwave powers of 20 mW, sweep widths of 100 G (centered at 3518 G), and modulation amplitudes of 1 G.

## 4. Conclusions

We demonstrated the effective chemical vapor deposition (CVD) synthesis of β-Cu_2−x_Se using only elemental precursors, such as Cu foil and Se powder, without requiring a reducing atmosphere. The resulting β-Cu_2−x_Se powder has been extensively investigated due to its potential as an ultra-fast photocatalyst. Post-cycling analyses using high-resolution scanning electron microscopy (HRSEM), high-resolution transmission electron microscopy (HRTEM), and X-ray photoelectron spectroscopy (XPS) have clearly shown its remarkable stability. With a rate constant of 3.14 × 10^−2^ min^−1^, the removal efficiency of TC-HCl could reach 98.36% in 90 min. In contrast, the ideal catalyst dosage was 20 mg, and the pH of the solution was 5.0. The active species trapping experiments demonstrated that the h^+^, ē, •O^2−^, and •OH were all involved in the photocatalytic reaction. The TC-HCl degrading efficiency was still able to reach 92.53% after four cycle runs, and the catalyst’s structures had not changed noticeably, indicating outstanding stability and durability. The results of this investigation suggest that β-Cu_2−x_Se has the potential to be used as a photoactive material in aquatic environments to oxidize antibiotic pollutants. This catalyst can be applied to different wastewater treatment procedures and the breakdown of other antibiotics.

## Data Availability

Data are contained within the article and Supplementary Materials.

## Supplementary Materials

The following supporting information can be downloaded at: https://www.mdpi.com/article/10.3390/molecules29040887/s1. Figure S1. HRSEM shows microstructural evolution and copper selenide (β-Cu_2−x_Se) formation at different reaction times (5mins to 3h) between etched Cu foil and elemental Se. Figure S2. HRSEM images, EDS, and elemental mapping of Cu and Se at different reaction times indicate the formation and evolution of β-Cu_2−x_Se. Figure S3. Proposed reaction mechanism based on the microstructural evolution and CVD-synthesis of β-Cu_2−x_Se on Cu foil. Figure S4. (a) Band gap and (b) Raman measurements of β-Cu_2−x_Se. Figure S5. Post-mortem studies showing (a) HRTEM image of β-Cu_2-x_Se after photocatalytic degradation (4 cycles); (b) SAED of post-cycled β-Cu_2−x_Se; (c) and (d) HRSEM images of bulk β-Cu_2−x_Se indicating that the material is very stable and does not undergo extreme microstructural changes after photocatalytic cycling (4 cycles). Figure S6. (a) and (b) are the XPS measurements of uncycled β-Cu_2−x_Se, while (c) and (d) are the XPS) measurements of cycled β-Cu_2−x_Se under simulated solar light. Figure S7. LC-MS of TCH degradation over β-Cu_2−x_Se under solar light irradiation. Figure S8. The mass spectra of TC-HCl transformation products: m/z = 467, 445, 427,410, 318, 274, 230. Table S1. Comparison of TC-HCl photodegradation using different photocatalysts. References [63,70,75,76,77,78,79,80,81,82,83] are cited in the Supplementary Materials.
