# Supplementary material for: Chemical-Vapor-Deposition-Synthesized Two-Dimensional Non-Stoichiometric Copper Selenide (β-Cu2−xSe) for Ultra-Fast Tetracycline Hydrochloride Degradation under Solar Light
Source: Molecules. 2024 Feb 17;29(4):887. doi: 10.3390/molecules29040887 (PMC10892667; doi:10.3390/molecules29040887)
Supplement: Supplementary file 1 [file molecules-29-00887-s001.zip › molecules-2865908-supplementary.pdf]

Supporting Information

Chemical-Vapor-Deposition-Synthesized Two-Dimensional Non-Stoichiometric Copper Selenide (β-$Cu_{2-x}Se$) for Ultra-Fast Tetracycline Hydrochloride Degradation under Solar Light

Srijith [1,†], Rajashree Konar [1,†], Eti Teblum [1], Vivek Kumar Singh [1], Madina Telkhozhayeva [1], Michelangelo Paiardi [2] and Gilbert Daniel Nessim [1,*]

[1] Department of Chemistry, Bar-Ilan Institute of Nanotechnology and Advanced Materials, Bar-Ilan University, Ramat Gan 5290002, Israel; srijith.nitk@gmail.com (S.); rajashree.konar@biu.ac.il (R.K.); eti.teblum@gmail.com (E.T.); vivekkumarsingh.rs.cer18@itbhu.ac.in (V.K.S.); telkhozhayeva@gmail.com (M.T.)

[2] Department of Chemistry and Materials Engineering "Giulio Natta", Politecnico Di Milano, Piazza Leonardo da Vinci, 32, 20133 Milano, Italy; michelangelo.paiardi@mail.polimi.it

* Correspondence: gilbert.nessim@biu.ac.il; Tel.: +972-373-84540

† These authors contributed equally to this work.

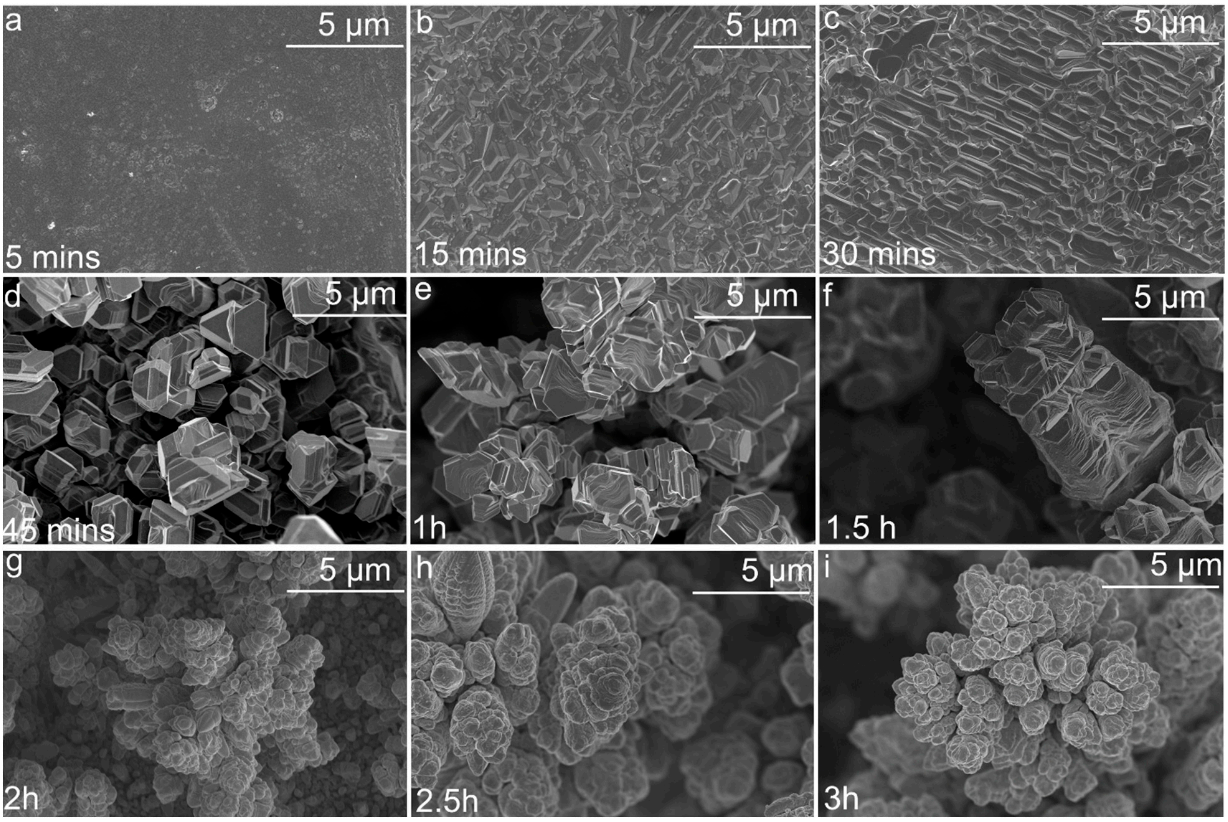

Figure S1. High-resolution scanning electron microscopy (HRSEM) shows microstructural evolution and copper selenide (β-$Cu_{2-x}Se$) formation at different reaction times (5mins to 3h) between etched Cu foil and elemental Se.

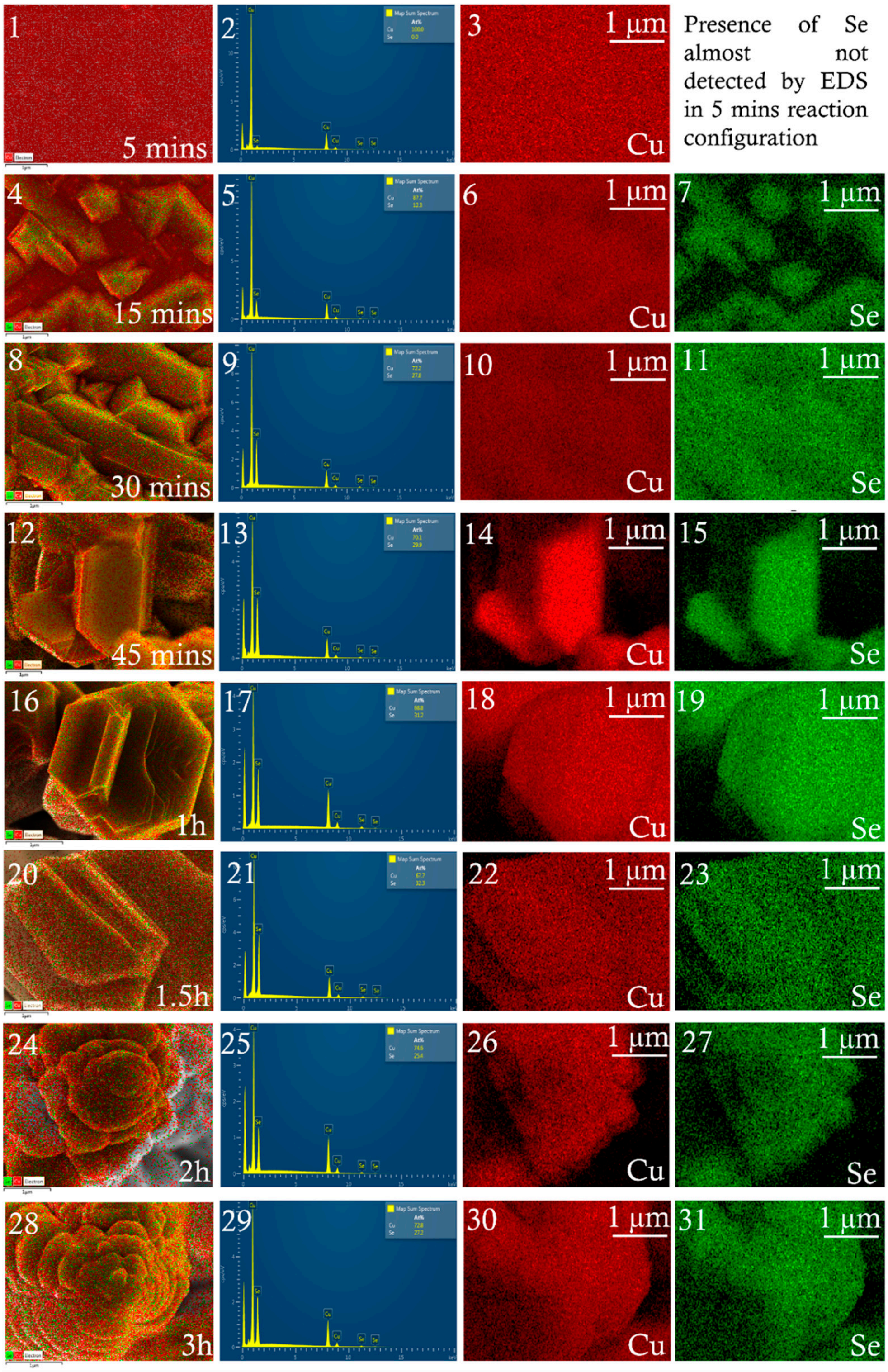

Figure S2. Ex-situ High-resolution scanning electron microscopy (HRSEM) images, Electron Dispersive Spectroscopy (EDS), and elemental mapping of Cu and Se at different reaction times indicate the formation and evolution of β-$Cu_{2-x}Se$.

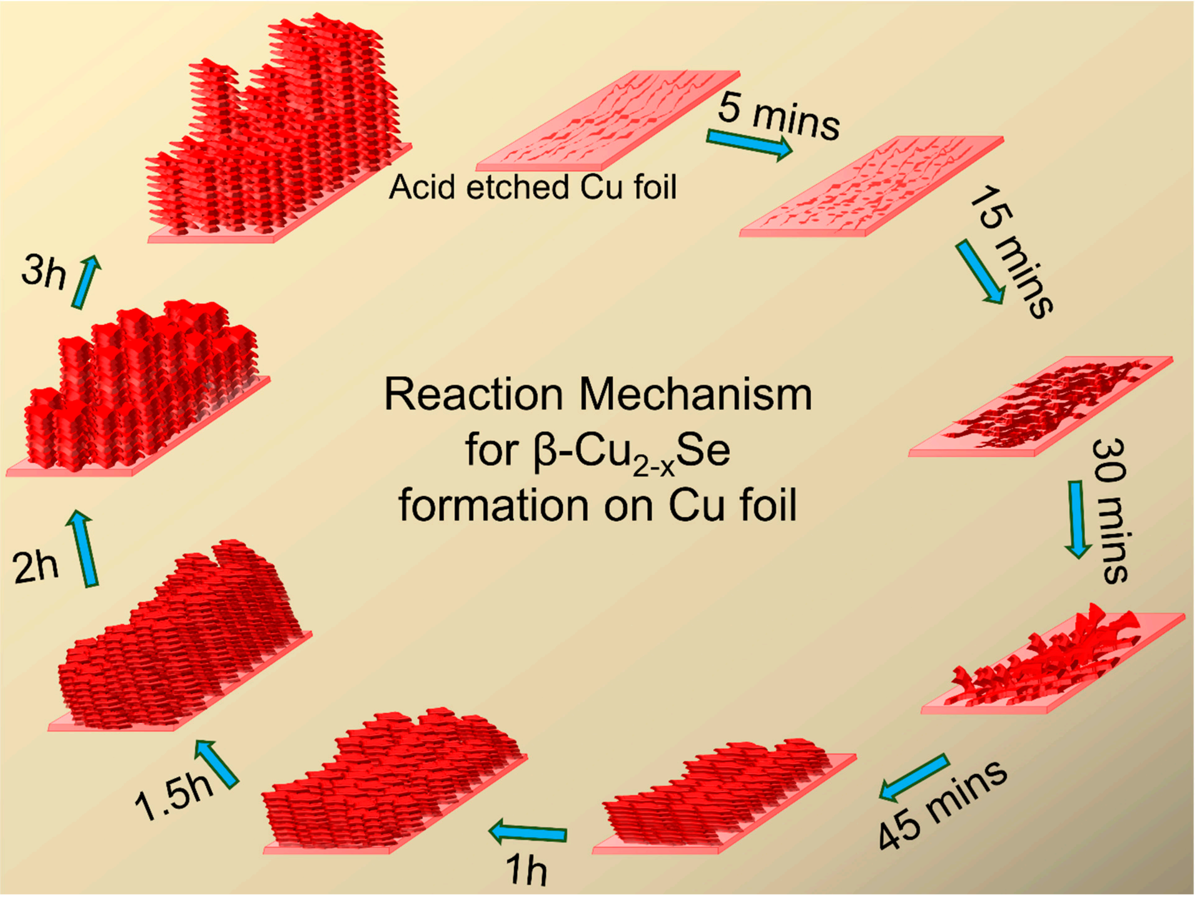

Figure S3. Proposed reaction mechanism based on the microstructural evolution and CVD-synthesis of β-Cu_{2-x}Se on Cu foil.

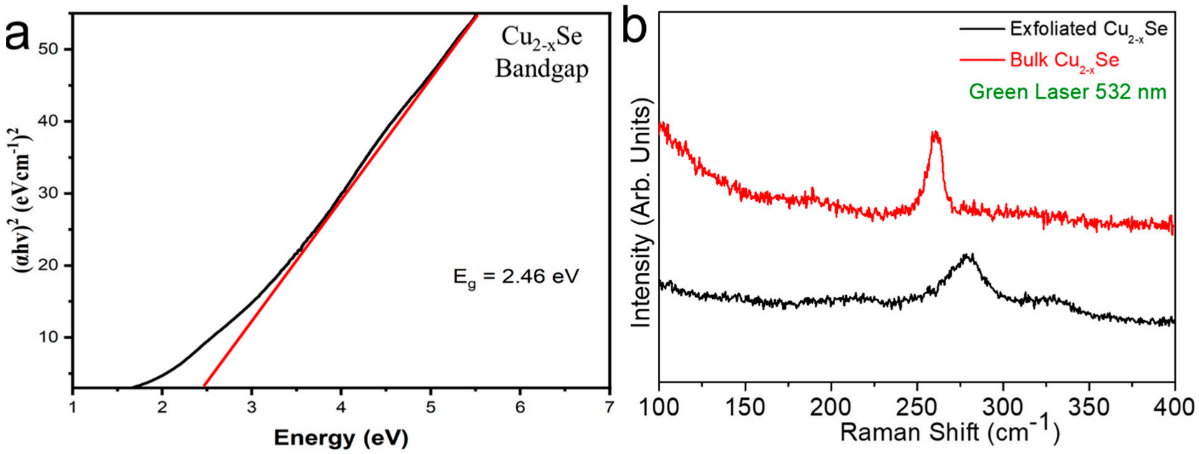

Figure S4. (a) Band gap and (b) Raman measurements of β-$Cu_{2-x}Se$.

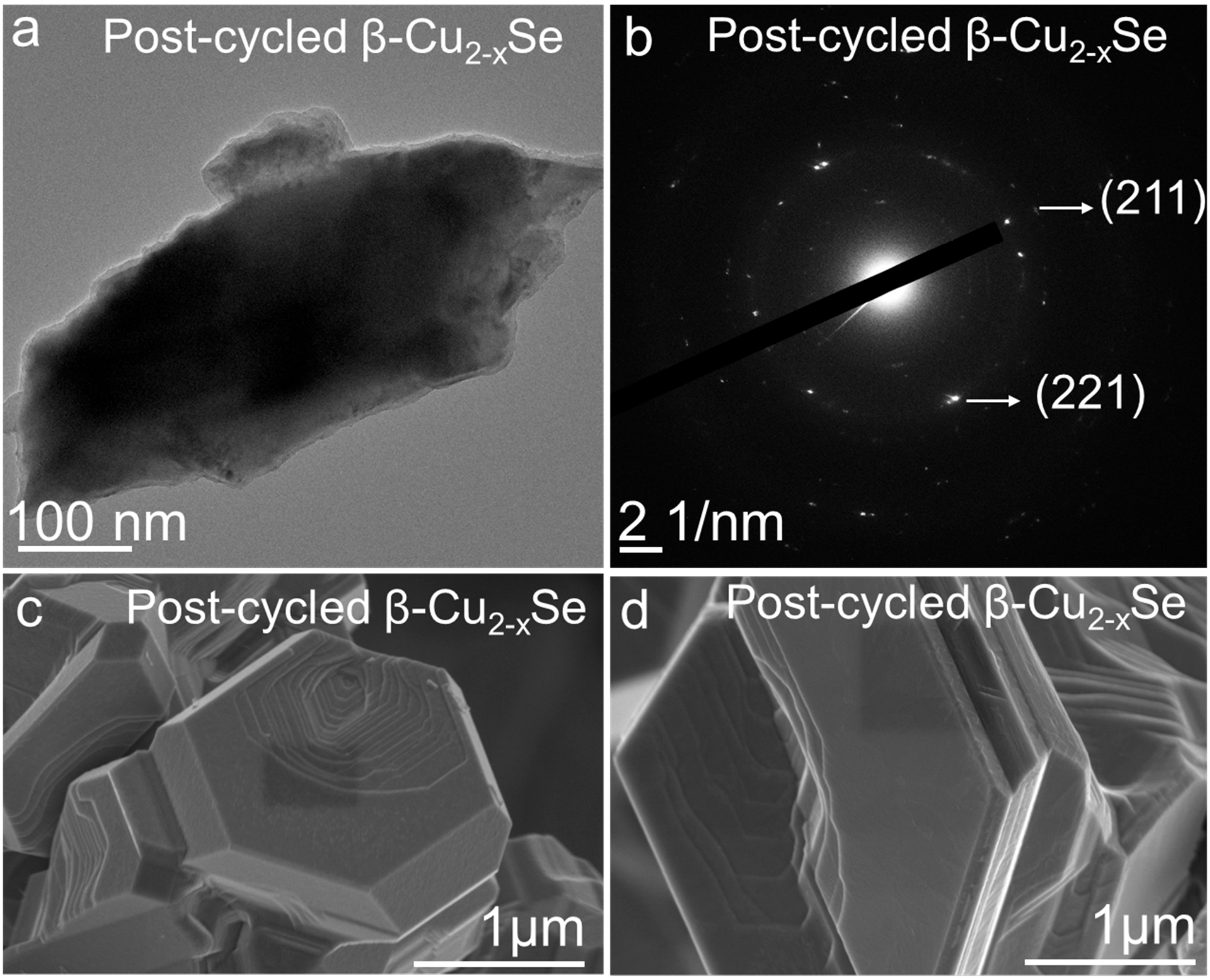

Figure S5. Post-mortem studies showing (a) High-resolution transmission electron microscopy (HRTEM) image of β-$Cu_{2-x}Se$ after photocatalytic degradation (4 cycles); (b) Selected Area Diffraction (SAED) of post-cycled β-$Cu_{2-x}Se$; (c) and (d) High-resolution scanning electron microscopy (HRSEM) images of bulk β-$Cu_{2-x}Se$ indicating that the material is very stable and does not undergo extreme microstructural changes after photocatalytic cycling (4 cycles).

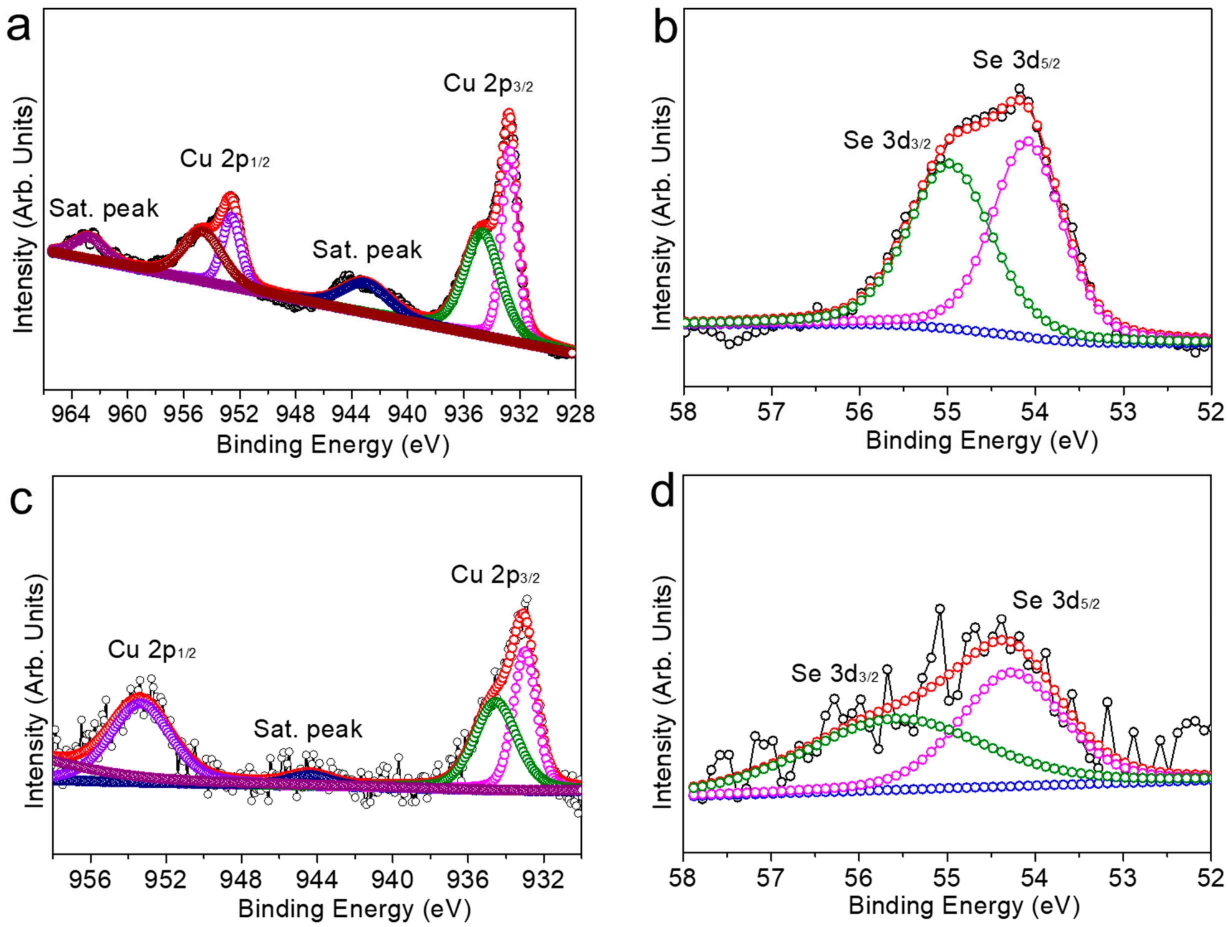

Figure S6. (a) and (b) are the X-Ray Photoelectron Spectroscopy (XPS) measurements of uncycled β-$Cu_{2-x}Se$, while (c) and (d) are the XPS) measurements of cycled β-$Cu_{2-x}Se$ under simulated solar light.

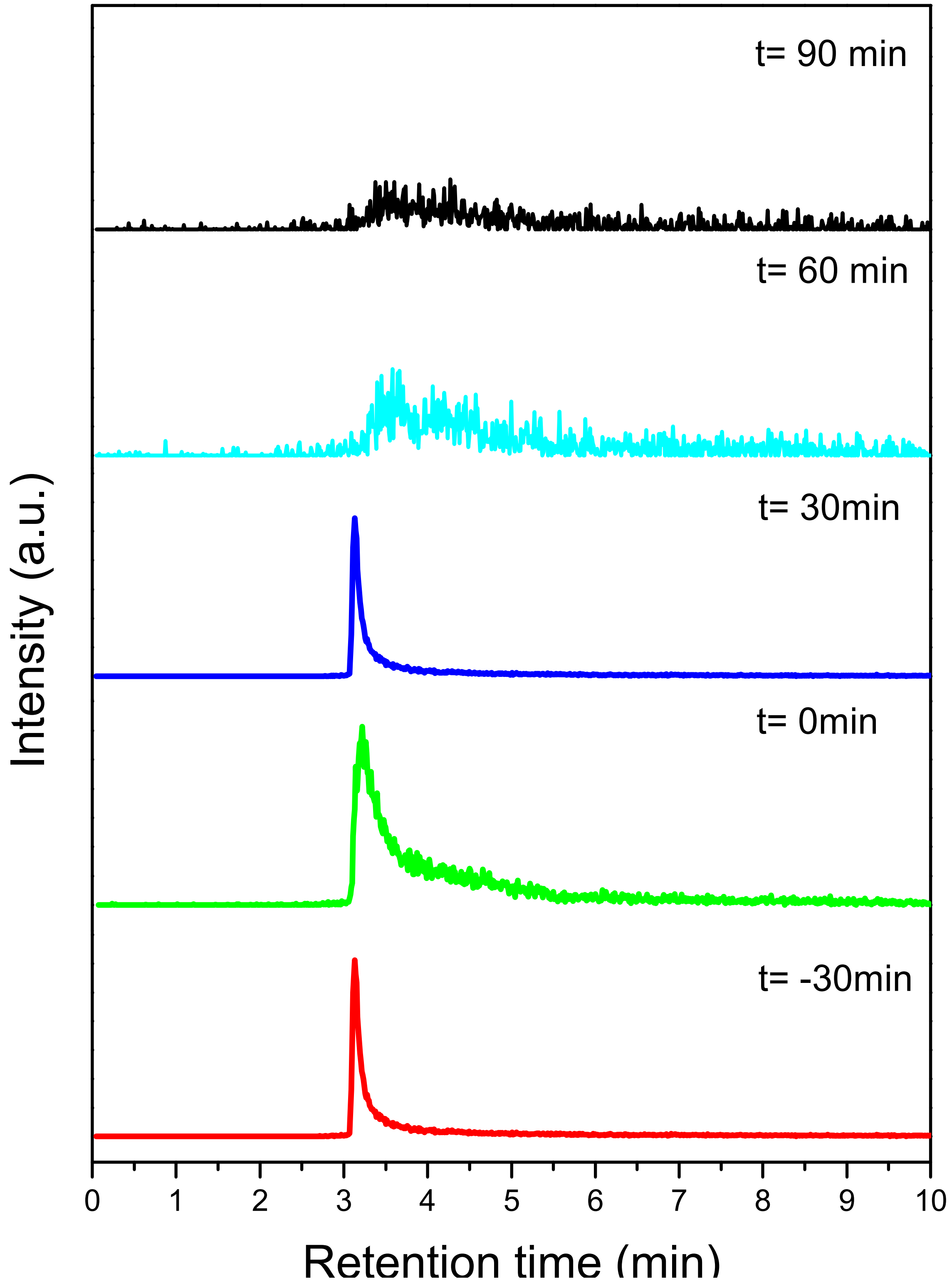

Figure S7. LC-MS of TCH degradation over β-$Cu_{2-x}Se$ under solar light irradiation.

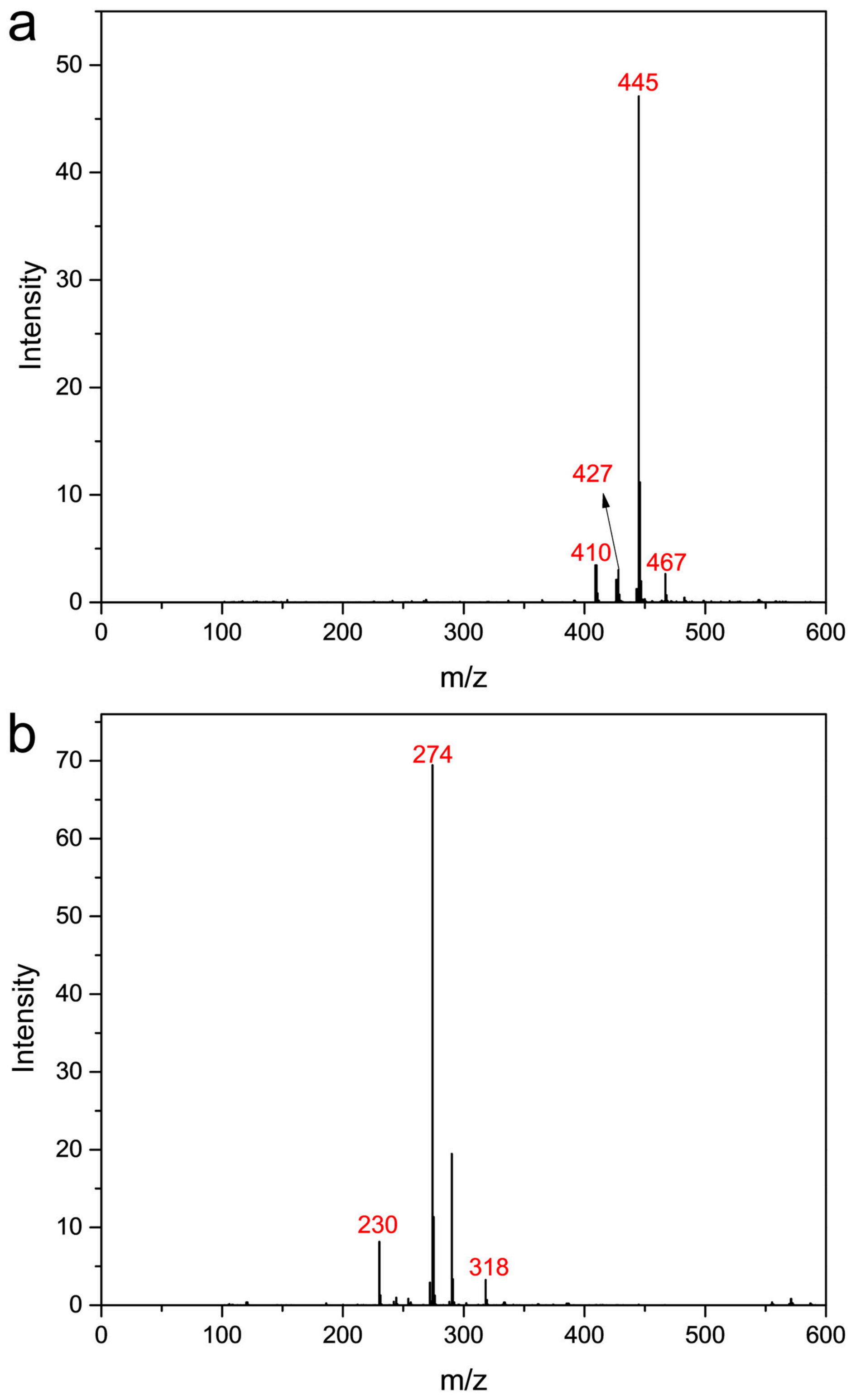

Figure S8. The mass spectra of TC-HCl transformation products: m/z = 467, 445, 427,410, 318, 274, 230.

Table S1. Comparison of TC-HCl photodegradation using different photocatalysts

Catalyst	Concentration of TC-HCl	Catalyst dosage	TC-HCl /Catalyst dosage	Degradation time	Efficiency	Reaction rate	Ref.
**β-$Cu_{2-x}Se$**	**20 mg/L**	**0.4 g/L**	**50 mg/g**	**90 min**	**98.35 %**	**0.0314 min^{-1}**	**Present Work**
TiS_2	20 mg/L	1 g/L	20 mg/g	300 min	95.43 %	0.0174 min^{-1}	[1]
Sn_3O_4/g-C_3N_4	10 mg/L	0.5 g/L	20 mg/g	120 min	72.2 %	0.0108 min^{-1}	[2]
CdS/Ti_{32}-oxo-cluster	50 mg/L	0.2 g/L	250 mg/g	60 min	96.3 %	0.06135 min^{-1}	[3]
$Bi_{24}O_{31}Br_{10}$	20 mg /L	0.3 g/L	67 mg/g	90 min	95 %	0.031 min^{-1}	[4]
WO_3/g-C_3N_4/Bi_2O_3	10 mg/L	1 g/L	10 mg/g	60 min	80.2 %	0.0236 min^{-1}	[5]
Ag/$AgIn_5S_8$	10 mg/L	0.3 g/L	33 mg/g	120 min	95.3 %	0.023 min^{-1}	[6]
BiOCl	10 mg/L	0.5 g/L	20 mg/g	90 min	71.8 %	0.0139 min^{-1}	[7]
WO_3/g-C_3N_4	20 mg/L	1 g/L	20 mg/g	60 min	90.54 %	0.0378 min^{-1}	[8]
CNFs/g-C_3N_4/BiOBr	20 mg/L	3 g/L	6.7 mg/g	120 min	86.1 %	0.015 min^{-1}	[9]
CDs/MoO3/g-C3N4	20 mg/L	0.6 g/L	33.3 mg/g	90 min	88.4 %	0.0231 min^{-1}	[10]
$ZnIn_2S_4$/MoO_3	30 mg/L	0.15 g/L	200 mg/g	90 min	94.5 %	0.0292 min^{-1}	[11]

References

1. Telkhozhayeva, M.; Hirsch, B.; Konar, R.; Teblum, E.; Lavi, R.; Weitman, M.; Malik, B.; Moretti, E.; Nessim, G.D. 2D TiS2 Flakes for Tetracycline Hydrochloride Photodegradation under Solar Light. *Appl. Catal. B Environ.* **2022**, *318*, 121872, doi:https://doi.org/10.1016/j.apcatb.2022.121872.

2. Li, C.; Yu, S.; Dong, H.; Liu, C.; Wu, H.; Che, H.; Chen, G. Z-Scheme Mesoporous Photocatalyst Constructed by Modification of Sn 3 O 4 Nanoclusters on g-C 3 N 4 Nanosheets with Improved Photocatalytic Performance and Mechanism Insight. *Appl. Catal. B Environ.* **2018**, *238*, doi:10.1016/j.apcatb.2018.07.049.

3. Zhu, Q.; Sun, Y.; Na, F.; Wei, J.; Xu, S.; Li, Y.; Guo, F. Fabrication of CdS/Titanium-Oxo-Cluster Nanocomposites Based on a Ti32 Framework with Enhanced Photocatalytic Activity for Tetracycline Hydrochloride Degradation under Visible Light. *Appl. Catal. B Environ.* **2019**, *254*, doi:10.1016/j.apcatb.2019.05.006.

4. Wang, C.; Zhang, X.; Qiu, H.-B.; Huang, G.; Yu, H.-Q. Bi24O31Br10 Nanosheets with Controllable Thickness for Visible–Light–Driven Catalytic Degradation of Tetracycline Hydrochloride. *Appl. Catal. B Environ.* **2017**, *205*, doi:10.1016/j.apcatb.2017.01.015.

5. Jiang, L.; Yuan, X.-Z.; Zeng, G.; Liang, J.; Chen, X.; Yu, H.; Hou, W.; Wu, Z.; Zhang, J.; Xiong, T. In-Situ Synthesis of Direct Solid-State Dual Z-Scheme WO 3 /g-C 3 N 4 /Bi 2 O 3 Photocatalyst for the Degradation of Refractory Pollutant. *Appl. Catal. B Environ.* **2018**, *227*, doi:10.1016/j.apcatb.2018.01.042.

6. Deng, F.; Zhao, L.; Luo, X.; Luo, S.; Dionysiou, D. Highly Efficient Visible-Light Photocatalytic Performance of Ag/AgIn 5 S 8 for Degradation of Tetracycline Hydrochloride and Treatment of Real Pharmaceutical Industry Wastewater. *Chem. Eng. J.* **2017**, *333*, doi:10.1016/j.cej.2017.09.022.

7. Yan, Y.; Tang, X.; Ma, C.; Huang, H.; Yu, K.; Liu, Y.; Lu, Z.; Li, C.; Huo, P.; Zhu, Z. 2D Mesoporous Photocatalyst Constructed by Modification of Biochar on BiOCl Ultrathin Nanosheets for Enhancing TC-HCl Degradation Activity. *New J. Chem.* **2019**, *44*, doi:10.1039/C9NJ05219D.

8. Pan, T.; Dongdong, C.; Xu, W.; Fang, J.; Wu, S.; Liu, Z.; Wu, K.; Fang, Z. Anionic Polyacrylamide-Assisted Construction of Thin 2D-2D WO3/g-C3N4 Step-Scheme Heterojunction for Enhanced Tetracycline Degradation under Visible Light Irradiation. *J. Hazard. Mater.* **2020**, *393*, 122366, doi:10.1016/j.jhazmat.2020.122366.

9. Shi, Z.; Zhang, Y.; Shen, X.; Duoerkun, G.; Zhu, B.; Zhang, L.; Li, M.; Chen, Z. Fabrication of G-C3N4/BiOBr Heterojunctions on Carbon Fibers as Weaveable Photocatalyst for Degrading Tetracycline Hydrochloride under Visible Light. *Chem. Eng. J.* **2020**, *386*, 124010, doi:10.1016/j.cej.2020.124010.

10. Zhijie, X.; Feng, Y.; Wang, F.; Chen, D.; Zhang, Q.; Zeng, Y.; Lv, W.; Liu, G. Construction of Carbon Dots Modified MoO 3 /g-C 3 N 4 Z-Scheme Photocatalyst with Enhanced Visible-Light Photocatalytic Activity for the Degradation of Tetracycline. *Appl. Catal. B Environ.* **2018**, *229*, doi:10.1016/j.apcatb.2018.02.011.

11. Ouyang, C.; Quan, X.; Zhang, C.; Pan, Y.; Li, X.; Hong, Z.; Zhi, M. Direct Z-Scheme ZnIn2S4@MoO3 Heterojunction for Efficient Photodegradation of Tetracycline Hydrochloride under Visible Light Irradiation. *Chem. Eng. J.* **2021**, *424*, 130510, doi:https://doi.org/10.1016/j.cej.2021.130510.
